# Epidemiology of compassion: A literature review

**DOI:** 10.3389/fpsyg.2022.992705

**Published:** 2022-11-17

**Authors:** David G. Addiss, Amy Richards, Sedem Adiabu, Emma Horwath, Sophie Leruth, Ashley L. Graham, Heather Buesseler

**Affiliations:** ^1^Focus Area for Compassion and Ethics (FACE), Task Force for Global Health, Decatur, GA, United States; ^2^Rollins School of Public Health, Emory University, Atlanta, GA, United States

**Keywords:** compassion, empathy, epidemiology, risk factor, psychology, public health, training, sociology

## Abstract

Psychology and neuroscience have contributed significantly to advances in understanding compassion. In contrast, little attention has been given to the epidemiology of compassion. The human experience of compassion is heterogeneous with respect to time, place, and person. Therefore, compassion has an epidemiology, although little is known about the factors that account for spatial or temporal clustering of compassion or how these factors might be harnessed to promote and realize a more compassionate world. We reviewed the scientific literature to describe what is known about “risk factors” for compassion towards others. Studies were included if they used quantitative methods, treated compassion as an outcome, and used measures of compassion that included elements of empathy and action to alleviate suffering. Eighty-two studies met the inclusion criteria; 89 potential risk factors were tested 418 times for association with compassion. Significant associations with compassion were found for individual demographic factors (e.g., gender, religious faith); personal characteristics (e.g., emotional intelligence, perspective-taking, secure attachment); personal experience (e.g., previous adversity); behaviors (e.g., church attendance); circumstantial factors during the compassion encounter (e.g., perceptions of suffering severity, relational proximity of the compassion-giver and -receiver, emotional state of the compassion-giver); and organizational features. Few studies explored the capacity to receive, rather than give, compassion. Definitions and measures of compassion varied widely across disciplines; 87% of studies used self-report measures and 39% used a cross-sectional design. Ten randomized clinical trials documented the effectiveness of compassion training. From an epidemiologic perspective, most studies treated compassion as an individual host factor rather than as transmissible or influenced by time or the environment. The causal pathways leading from suffering to a compassionate response appear to be non-linear and complex. A variety of factors (acting as effect modifiers) appear to be permissive of—or essential for—the arising of compassion in certain settings or specific populations. Future epidemiologic research on compassion should take into account contextual and environmental factors and should elucidate compassion-related dynamics within organizations and human systems. Such research should be informed by a range of epidemiologic tools and methods, as well as insights from other scientific disciplines and spiritual and religious traditions.

## Background

Compassion is a response to suffering that involves cognitive awareness, empathy, and action to alleviate suffering. Psychology and neuroscience have contributed significantly to advancing the understanding of compassion in recent years. In contrast, relatively little attention has been given to the epidemiology of compassion. Epidemiology, the quantitative science that informs public health, is used to describe how and why phenomena are clustered in terms of time, place, and person; to identify causal relationships; to develop metrics and apply them for monitoring and evaluating interventions; and to provide evidence for policy and advocacy. Typically, epidemiology has focused on disease, injury, and other threats to human health. By identifying “risk factors,” i.e., variables associated with increased likelihood of a disease (or other outcome of interest), epidemiologists can help to determine what causes that disease and promote behaviors and policies to prevent it.

VanderWeele and colleagues recently highlighted the need for a “positive epidemiology” that aligns with the field of positive psychology, “a positive epidemiology that takes as its object not only disease but also health in its fullest sense” (VanderWeele et al., 2020). Despite pioneering work by Levin and others on the epidemiology of love (Levin, 2000, 2022), the field of positive epidemiology remains under-developed.

The character strength of compassion, valued by all major world religions and spiritual traditions, is essential to human society (Armstrong, 2009). In general, humans experience compassion as “clustered”—we do not experience compassion at the same level of intensity and quality at all times, in all places, and from all people. Therefore, compassion has an epidemiology, although little is known from a quantitative perspective about how compassion is distributed or about the most effective ways to foster compassion in different stages of life, specific populations, or environments.

Understanding the epidemiology of compassion could have practical significance. The lack of compassion in current social discourse, fueled by political polarization and the trauma of the COVID-19 pandemic, is of increasing concern. The past two decades have witnessed an explosion of interest in loving-kindness and compassion meditation, as well as other forms of contemplative practice to foster mindfulness and resilience. A growing body of scientific evidence demonstrates the effectiveness of such practices at the individual level (Riess et al., 2012; Jazaieri et al., 2013; Brito-Pons et al., 2018; Gonzalez-Hernandez et al., 2018), but little is known about how to effectively “scale up” compassion to the organizational or population levels.

Trzeciak and Mazzarelli (2019) recently documented the benefits of compassion for patient outcomes, physician well-being, and hospital systems, and compassion is increasingly recognized as essential for quality healthcare (Ghebreyesus, 2018). Several countries, including Scotland, Ethiopia, and Malaysia have highlighted compassionate care in their national health plans (The Scottish Government, 2010; Federal Democratic Republic of Ethiopia Ministry of Health, 2015; Ministry of Health Malaysia, 2021). However, current knowledge is insufficient to make detailed, evidence-based recommendations for developing compassionate health systems, and validated metrics to monitor progress on compassionate care within these systems are lacking. Providing such evidence is the purview of epidemiology.

The many different views of compassion represent a challenge for epidemiology, which requires clear, quantifiable case definitions. Gilbert, in particular, has explored controversies about the nature and origins of compassion (Gilbert, 2017, 2020). Some investigators define compassion in terms of its constituent components (Goetz et al., 2010; Strauss et al., 2016; Worline and Dutton, 2017; Gu et al., 2020). Others regard compassion primarily as a feeling or emotion, a motivation, or a disposition (Goetz and Simon-Thomas, 2017). Still others focus on the role of intention and self-related goals in moving from deliberation to compassionate action (Poulin, 2017; Gilbert, 2020).

As global health practitioners, our working understanding of compassion reflects the practical, action-oriented nature of the field. We view compassion as having the three essential elements (not necessarily sequential) of awareness (cognitive appraisal), empathic resonance, and action to relieve and prevent suffering (Focus Area for Compassion and Ethics, 2022). We agree with Gilbert and others that compassion extends beyond an immediate response to suffering to include prevention, avoidance of harm, and promotion of human flourishing (Gilbert and Choden, 2013; Gilbert, 2020). For the purposes of this review, our case definition of compassion required evidence of empathy and either action or the intention to act to alleviate suffering or distress.

## Materials and methods

We conducted a detailed review of the literature to identify risk factors for compassion (i.e., factors that have been quantitatively associated with compassion). We were broadly interested in other-directed compassion (i.e., compassion directed toward other humans, rather than oneself) and compassion as an *outcome* (not as a predictor of other potential benefits, such as improved health). We searched the available literature through April 2021 in the following subject areas: healthcare, psychology, sociology, anthropology, religion and faith, early childhood development, education, business, organizational development, mindfulness training, contemplative studies, communications, arts, and government.

Studies were *included* in the analysis if they reported quantitative findings and used a measure of compassion that included empathy and either action or the intention to act to alleviate suffering or distress. Studies were *excluded* from the analysis if the authors used only qualitative methods, reported only qualitative results, or if the measure of “compassion” was limited to empathy (emotional resonance) without action or intention to act. Studies that focused on prosocial behavior or altruism, including those involving “money games,” were not included unless they were explicitly situated within a context of suffering or distress, and the authors’ intent to study compassion was evident. Similarly, studies that focused on self-compassion as an outcome were not included, as our interest was in giving or receiving other-directed compassion. Articles that addressed “compassion satisfaction” and so-called “compassion fatigue” as outcomes were also excluded.

**Figure 1:**
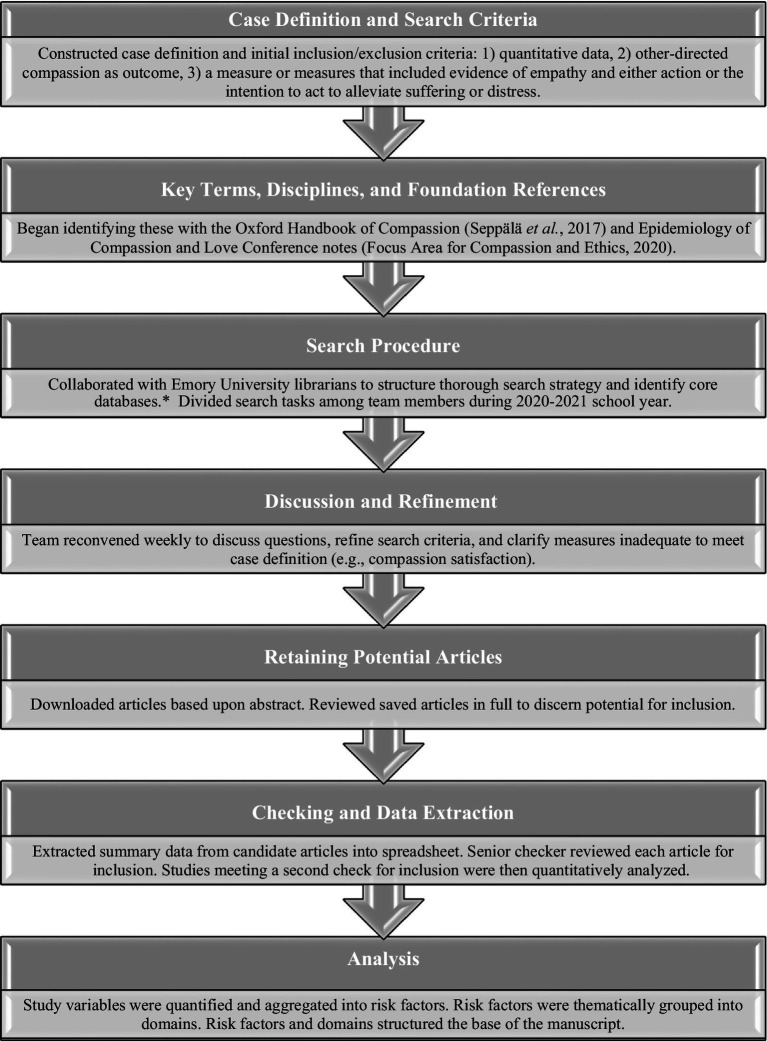
Schematic illustration of work flow. *Databases searched included PubMed, EBSCO (PsychInfo, SocINDEC, Academic Search Complete, Encyclopedia of Religion and Philosophy), JSTOR, Scopus, Web of Science, Sociological Abstracts, Social Sciences Full Text, CAB Direct, and Google Scholar.

A search strategy and terms were developed for each subject area (Figure 1), guided in part by the Oxford Handbook of Compassion Science (Seppälä et al., 2017) and proceedings of a January 2020 symposium on the epidemiology of compassion and love (Focus Area for Compassion and Ethics, 2020). Specific areas were assigned to students pursuing their masters’ degree in public health at Rollins School of Public Health and staff members of the Focus Area for Compassion and Ethics (FACE). Relevant databases (Figure 1) were searched for articles on compassion. The abstract of each article was reviewed, and if deemed potentially relevant, the full article was reviewed for inclusion in the analysis. The team met weekly during the 2020–2021 academic year to discuss preliminary findings, refine criteria for inclusion, cross-check references, and resolve issues.

Relevant articles were reviewed by three team members, who extracted information into a spreadsheet. For each potential risk factor, the direction of association with compassion (positive, negative, or no significant effect) was noted, as well as whether the risk factor was regarded as an independent variable or an effect modifier. For each study, other characteristics were also recorded, including age and gender of subjects; definitions of compassion and of risk factors, as well as the measures used to assess them; whether compassion was considered a state, trait, or skill; level of assessment (individual, organizational, or community); study design; and analytic method. Information was recorded on whether compassion was assessed from the perspective of the compassion-giver (“first-person” measure), the receiver of compassion (“second-person” measure), or an independent observer (“third-person” measure; Mascaro et al., 2020).

Risk factors for each article were assigned to one of four categories:

*Associated.* Having a statistically significant independent association with compassion in the population or a sub-population studied.
*Not associated.* Having no statistically significant independent association with compassion.
*Effect modifier.* Significantly modifying the direct relationship between other risk factors and compassion, for example, gender in a study of empathy training in which compassion scores improved among women, but not men (Riess et al., 2012).
*Second-order modifier.* Significantly modifying relationships among other risk factors that were themselves associated with compassion. For example, previous experience of adversity modifies the relative strength of a compassionate response to suffering of individuals vs. larger groups (Lim and DeSteno, 2020).

After potential risk factors were identified, we used an iterative process to group them into six interrelated themes or domains. This grouping helped to shape further exploration and facilitated comparison with the three main parameters of descriptive epidemiology: person, time, and place.

## Results

### Study characteristics

More than ten thousand articles were captured by search terms and reviewed for relevance. Sixty-four articles met the criteria for inclusion in the analysis. Of these, 44 (68.8%) articles came from the fields of psychology, sociology, anthropology, or childhood development; 14 (21.9%) articles addressed compassion in healthcare settings; 13 (20.3%) evaluated training or immersion programs to improve compassion, mindfulness, or empathy; and 10 (15.6%) involved organizational dimensions of compassion. These categories are not mutually exclusive (e.g., some articles assessed compassion training in healthcare settings).

These 64 articles reported results of 82 separate studies. Of these studies, 32 (39.0%) were cross-sectional in design (mostly surveys) and 25 (30.5%) were randomized experiments or clinical trials (RCTs); 14 (17.1%) studies evaluated interventions without randomization or control groups; 7 (8.5%) followed cohorts longitudinally but did not test interventions; 2 (2.4%) employed experience sampling methods; and 2 (2.4%) were meta-analyses (Table 1). None of the individual 41 studies in the meta-analysis by Butts et al. (2019) were included in our review. Only one of the 64 studies in the other meta-analysis, by Howick et al. (2017), which used the Consultation and Relational Empathy (CARE) measure to assess empathy of medical practitioners, was included in our review as a separate article. This study (Lelorain et al., 2015) included compassion-related measures and examined risk factors other than those reported by Howick et al. (2017).

**Table 1:** Characteristics of 82 studies included in the analysis of risk factors for compassion.

Characteristic	Value	No. (%) of studies
Study design	Survey (cross-sectional)	32 (39.0)
	Randomized clinical trials or experiments	25 (30.5)
	Interventions without randomization or control groups	14 (17.1)
	Longitudinal cohorts, without intervention	7 (8.5)
	Experience sampling	2 (2.4)
	Meta-analysis	2 (2.4)
Assessment perspective (who assessed compassion?) *	1^st^ person—self-report of the potential giver or agent of compassion	71 (86.6)
	2^nd^ person—assessment by the potential receiver or target of compassion	9 (11.0)
	3^rd^ person—objective or behavioral measure of compassion	20 (24.4)
Source of compassion (compassion-giver)	Individual only	79 (96.3)
	Organization only	1 (1.2)
	Individual and organization	2 (2.4)
Target of compassion (compassion-receiver) **	Hypothetical individual	50 (61.0)
	Actual individual	30 (36.6)
	Hypothetical group	41 (50.0)
	Actual group	16 (19.5)
	Environment (the earth)	1 (1.2)
Compassion considered as	Trait	65 (79.3)
	State	24 (29.3)
	Skill	17 (20.7)

*18 studies used >1 perspective.**Some measures include both individuals and groups.

Researchers used a variety of measures to assess compassion (Table 2). By far the most common approach was self-report of the person being evaluated for their tendency or capacity to give compassion to others—the “compassion-giver” (i.e., first-person measure); 71 (86.6%) studies included at least one such self-report measure. The validated first-person self-report scales most commonly used were Compassionate Love Scale for Humanity (Sprecher and Fehr, 2005) in eight studies and the Santa Clara Brief Compassion Scale (Huang et al., 2008) in seven studies. Investigators in 23 studies asked subjects to rate their feelings of compassion, usually in combination with other measures, while 15 studies assessed self-reported willingness to help, usually in combination with other measures. In nine (11.0%) studies, compassion was assessed by the potential receiver (“target”) of compassion (i.e., second-person measure). The most commonly used second-person measure was the CARE scale (Mercer et al., 2004; 4 studies; Table 2). Twenty (24.4%) studies used an objective measure of behavior to assess compassion (third-person measure), including offering to donate money (9 studies) or rendering assistance (5 studies) to a person in distress, usually in experimental settings. These categories are not mutually exclusive.

**Table 2:** Measures used to assess compassion in 82 studies examining compassion as an outcome.

Perspective	Scale or measure	No. studies
1^st^ person (self-report)	Feelings of compassion for person(s) suffering, i.e., victim(s) or patient(s)	23*
	Santa Clara Brief Compassion Scale (Huang et al., 2008)	7
	Compassion Scale, Pommier (Pommier et al., 2020)	6
	Dispositional Positive Emotions Scale (DPES; Shiota et al., 2006)	6*
	Compassionate Love scale—strangers and humanity (Sprecher and Fehr, 2005)	8
	Compassionate Love scale—close others (Sprecher and Fehr, 2005)	4
	Compassionate Love scale—specific others (Sprecher and Fehr, 2005)	2
	Self-reported willingness to help	15*
	Temperament and Character Inventory (TCI)—compassion subscale (Clonninger et al., 1993)	3
	Fears of Compassion Scales (Gilbert et al., 2011)	3
	Interpersonal Reactivity Index (IRI; Davis, 1983)	3*
	Abbreviated Compassionate Love scale (Krause and Hayward, 2015; Krause et al., 2018)	2*
	Self-Other Four Immeasurables scale (SOFI; Kraus and Sears, 2009)	1
	Questions from Monitoring the Future Survey (National Institute on Drug Abuse, 2022)	1
	Compassion of Others’ Lives (COOL; Chang et al., 2014)	1
	Prosocial Tendencies Measure-Revised (Carlo and Randall, 2002)	1*
	Amount of money the theoretical “victim” should receive from social welfare (Delton et al., 2018)	2*
	Compassion Engagement and Action scales—for others (Gilbert et al., 2017)	1
	Self-report caring behaviors (Jazaieri et al., 2016)	1
	Questionnaire for Cognitive and Affective Empathy (Reniers et al., 2011; Runyan et al., 2019)	1*
	Compiled measure of empathy, compassion and recent helping behavior (Runyan et al., 2019)	1
	Environmental Motives Scale (Bengtsson et al., 2016)	1
2^nd^ person (target of compassion)	Consultation and Relational Empathy Scale (CARE; Mercer et al., 2004)	4
	Schwartz Center Compassionate Care scale (Rodriquez and Lown, 2019)	2
	Patient ratings of physician’s compassion (similar to CARE)	1
	Compassionate affection scale—Shaver et al. (Shaver et al., 1987; Eldor, 2018)	1
	Compassion Engagement and Action scales—from others (Gilbert et al., 2017)	1
	Frequency or quality of compassion received (Moon et al., 2014; Sabey and Rauer, 2018)	2
3^rd^ person (behavioral measures)	(Amount of) money willing to donate	9*
	Offering assistance to someone in need or distress	5*
	Time spent helping confederate (Lim et al., 2015; Lim and DeSteno, 2016)	2*
	Carkhuff Empathy Scale (Carkhuff, 1969; Bas-Sarmiento et al., 2019)	1
	Reynolds Empathy Scale (Reynolds, 2000)	1
	Peer nomination: “shows strong compassion for others” (Bengtsson et al., 2016)	1
	Content analysis of Tweets (Boulianne et al., 2018)	1
	Emotion Recognition Index (Scherer and Scherer, 2011)	1*
	Healthcare provider’s rating of their team and their organization (Lown et al., 2020)	1
	Compassion Scale, Pommier (4 items modified for observer rating; McDonald et al., 2018)	1
	Psychologists’ rating of participants’ recorded responses to stories of personal distress (Palgi et al., 2015)	1

*At least one study paired this measure with another measure to create a full measure of compassion.

Potential “actors” or sources of compassion (compassion-givers) were individual people in 81 (98.8%) studies, an organization in three (3.7%) studies, and both individuals and an organization in two (2.4%) studies (Table 1). The self-report measures completed by individual compassion-givers tend to refer to receivers (targets) of compassion in a hypothetical or general sense, although some experimental studies assessed compassion towards real persons (e.g., patients, actors, or confederates whose role was part of the study design). Potential recipients of compassion were hypothetical individuals or groups in 50 (61.0%) and 41 (50.0%) studies, respectively, actual individuals or groups in 30 (36.6%) and 16 (19.5%) studies, and the environment in one (1.2%) study. Eight (9.8%) studies assessed compassion using both hypothetical and actual persons.

Sixty-five (79.3%) studies treated compassion as a trait (i.e., a stable personality characteristic). Twenty-four (29.3%) regarded compassion as a state (i.e., a short-term pattern of thought or behavior). Seventeen (20.7%) studies treated compassion as a skill (Table 1). Some studies considered compassion in more than one of these categories.

Demographic information on study subjects was incomplete. More than 82,000 subjects were studied. Among the 71 studies that reported participant gender, the proportion of females ranged from 30 to 100% (mean 61.5%). All but two studies, both meta-analyses, reported participant age range. Sixty-five (81.3%) studies included young adults (ages 18–29 years), most often university students. Five (6.3%) studies included children less than 18 years old, 46 (57.5%) included persons 30–60 years of age, and 33 (41.3%) included older adults. Mean age of subjects in each study ranged from 13 to 77 years. Race and ethnicity were often not recorded. Of the 82 studies, 15 (18.3%) were conducted entirely in Western Europe and 46 (56.1%) in North America. Four (4.9%) studies were conducted entirely in India (Choudhary and Madnawat, 2017a,b; Singh et al., 2018; Prabha and Mittal, 2019), two (2.4%) each in Israel (Eldor, 2018; Prabha and Mittal, 2019) and Chile (Brito-Pons et al., 2018), and one (1.2%) each in Malaysia (Owuamalam and Matos, 2019) and South Korea (Moon et al., 2014). Eleven (13.4%) additional studies used data from multiple countries, including countries in Western Europe and North America, Israel, Turkey, nine countries in South America (Chang et al., 2021), and Ethiopia, China, and Japan (Howick et al., 2017).

### Risk factors

A total of 89 potential risk factors for compassion were identified and categorized into six themes or domains to facilitate further analysis.

- Domain 1—Demographic features (mostly of the compassion-giver)
- Domain 2—Personal characteristics, including disposition and skills of the compassion-giver
- Domain 3—Personal history and experience of the compassion-giver
- Domain 4—Habitual behaviors of the compassion-giver
- Domain 5—Circumstantial or contextual factors of the “compassion encounter,” when compassion is given or withheld
- Domain 6—Organizational or structural characteristics

**Figure 2:**
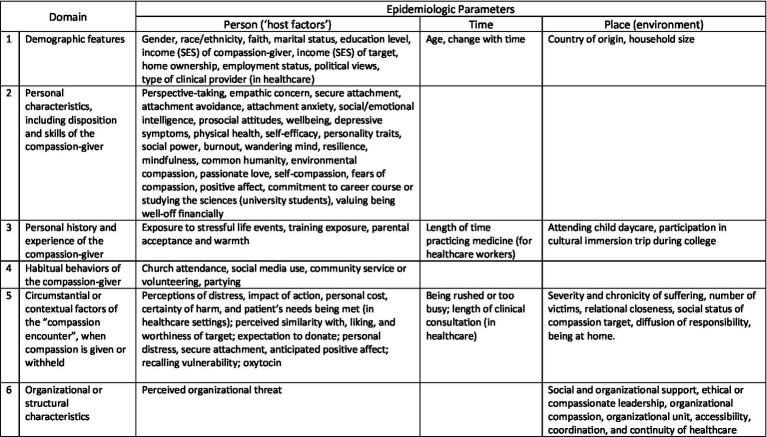
Primary alignment of potential risk factor domains with the epidemiologic parameters of person, time, and place. Even though individual potential risk factors are listed only once, some risk factors may be active in multiple domains and affect multiple parameters, e.g., environmental disasters are stressful personal life events. Risk factors refer to the compassion-giver unless otherwise noted.

The 89 potential risk factors were tested a total of 418 times for association with other-directed compassion; 56 (68.3%) potential risk factors were assessed in more than one study. The vast majority of risk factors referred to demographic features and personal characteristics of individual persons, i.e., host factors, as well as to circumstantial factors at the moment of the compassion encounter (Figure 2).

Potential risk factors assessed for association with compassion (either as independent risk factors or effect modifiers) are summarized in Table 3. Among potential risk factors that were evaluated in three or more tests of association and found to be associated with compassion in ≥50% of those tests, *demographic factors* included female gender (51% of tests of association being positively associated with compassion), religious faith (77%), socioeconomic status of the compassion-receiver (50%), and country of origin. Factors positively related to *personal characteristics* included social and emotional intelligence (100%), prosocial attitudes and values (100%), personal well-being or eudaimonia (100%), personality traits of openness (80%) and humility (67%), self-compassion (80%), the capacity for perspective-taking (80%), secure emotional attachment (75%), and empathic concern (71%). Attachment avoidance was negatively associated with compassion (91% of tests of association).

**Table 3:** Potential risk factors evaluated, by domain, and direction of statistically significant association with compassion as an outcome.*

	Variable		No. Studies	No. Assoc.	Positive association			No effect			Negative association			Modifier of modifier	
					Independent risk factor		Effect modifier	Independent risk factor		Effect modifier	Independent risk factor		Effect modifier	Some Effect	No Effect
					Overall	Some strata		Overall	Some strata		Overall	Some strata			
**Domain 1**															
Demographic features	Age (increasing)		17	17	4			9		2	2					Gender (female = 1)		41	47	23	1		19		2	1			1		Race/ethnicity		7	7	2			5								Race/ethnicity (compassion target)		2	2	2											Religiosity/spirituality/faith		10	13	10			3								Country of origin/study (any difference)		9	10	9		1									Marital status (married = 1)		5	6				5			1					Education level		6	6	2			2		1	1					Income (SES) of compassion-giver		8	9	1			3		1	3		1			Income (SES) of compassion-target		4	4	2			1			1					Home ownership		2	2	1			1								Household size		1	1				1								Employment status		1	1				1								Politically liberal		2	2	1									1		Clinical provider type (any difference)	3	3	1			2							
**Domain 2**															
Personal characteristics (including disposition and skills)	Perspective taking		7	10	6		2	1						1		Empathic concern		12	24	13		4		5	1					1	Secure attachment (dispositional)		2	4	3			1								Attachment avoidance		9	11				1			9	1				Attachment anxiety		8	11				9	1			1				Self-compassion		5	5	4			1								Social and emotional intelligence		3	3	3											Prosocial attitudes or values		3	3	3											Well-being/eudaimonia		2	4	3		1									Depressive symptoms		4	5				3			1		1			Physical health		4	5	1			1			1	1	1			Efficacy (self-efficacy)		4	6	2					1				3		Personality – humility		2	3	1	1			1							Personality – openness		4	5	4			1								Personality – conscientiousness	3	5	1	1		2	1						
	Personality – extraversion		3	4	1			3							
	Personality – neuroticism		4	5				5							
	Personality – agreeableness		3	5	1	1		2	1						
	Personality – emotionality		1	1	1										
	Social power (of compassion-giver)		1	2							1		1		
	Burnout		2	2				1			1				
	Mind wandering to negative		1	1							1				
	Resilience		1	1	1										
	Mindfulness		1	1						1					
	Common humanity		1	1	1										
	Environmental compassion		1	1	1										
	Passionate love		1	1	1										
	Fears of compassion		1	1							1				
	Positive affect (in compassion-giver)		1	1				1							
	Committed to a career course		2	2	2										
	Studying the natural/social sciences		2	2	1			1							
	Valuing being financially well-off		1	1							1				
**Domain 3**															
Personal history or experience	Exposure to stressful life events		9	13	7	2								4		Training exposure		14	15	12			3								Parental acceptance		1	1	1			1								Parental warmth		1	1	1											Attending child daycare		1	2	1			1								Cultural immersion trip during college		1	1	1											Length of time practicing medicine		1	1	1										
**Domain 4**															
Habitualbehaviors	Church attendance		5	7	4			3								Social media use		1	1	1											Doing community service/volunteering		2	3	1	1						1				Partying behavior		2	2							2				
**Domain 5**																													
Circumstantial or contextual factors related to the compassion encounter	Perception of suffering & outcome	Severity of suffering		4	6	4		1							1		Chronicity of suffering	1	2				1		1						Number of “victims”	6	11		4			2		1	2		2		Perceived distress in target	2	3	1	1	1									Perceived positive impact of compassionate action	1	1	1											Perceived personal cost of compassionate action	1	2							1			1		Perceived certainty of harm	1	1										1		Patient’s care needs are met	2	2	2										
	Relational aspects	Perceived similarity/in-group	6	8	8											Liking/valuing other	2	2	1			1								Relationship closeness/proximity	6	6	5			1								Expectation to donate	1	2					1			1				High social status (target)	2	2		1		1								Perceived worthiness	1	1			1									Diffusion of responsibility	1	1						1					
	Inner state		Personal or empathic distress	15	18	5	1		4		3		2	2	1		Secure attachment (situational)	7	9	6		2	1								Anticipated positive affect	3	5	2									3		Recalling vulnerability	2	3				2				1			
	Time			Being rushed/too busy	1	1							1					Length of clinical consultation	2	2	2										
	Other				Oxytocin	1	1		1										Being at home	1	1	1										
**Domain 6**Organizational and structural characteristics																														Social and organizational support		3	3	3											Ethical or compassionate leadership or management		3	3	3											Organizational compassion		3	3	3											Perceived organizational threat		2	2				1			1					Organizational unit		1	1	1											Healthcare accessibility (organizational)		1	1	1											Continuity of care		1	1	1											Coordination of care		1	1	1										

* Variables refer to the compassion-giver unless otherwise noted.

Factors related to *personal history* included participation in compassion, empathy, or mindfulness training (80%) and previous exposure to stressful life events (69%). Among *habitual behaviors*, 57% of tests of church attendance were significantly associated with compassion. Participation in community service or volunteering also was associated with compassion (67%).

*Circumstantial factors* significantly associated with compassion in ≥50% of studies that examined them included in-group similarity (100%), perceived distress in the target, i.e., the person suffering (100%), a sense of secure attachment in the compassion-giver (89%), perceived severity of suffering (83%), and relational closeness between the compassion-giver and the target (83%).

*Organizational or structural factors* associated with compassion in ≥50% of studies that examined them included social or organizational support, ethical or compassionate leadership, and organizational compassion. Each of these variables was examined in three separate studies, all of which showed a positive association with individual-level compassion within the organization.

Risk factors are described below in more detail and shown in Table 3. Except where noted, risk factors refer to the compassion-giver rather than the recipient of compassion.

#### Domain 1—Demographic features

##### Age (17 studies, 17 tests of association)

The relationship between compassion and age was mixed, with 11 of 17 tests showing no association. In many studies, particularly those involving college students, age range was limited. However, two longitudinal cohort studies reported that compassion increased between 30 and 50 years of age (Hintsanen et al., 2019; Saarinen et al., 2020). In contrast, Sabey and Rauer (2018) found that self-reported compassionate love for others declined over a 17-month period among older heterosexual married couples (mean age 71 years). Bengtsson et al. observed a decrease in compassion for others in adolescents between 12 and 14 years of age. This decline was linked to negative self-perceptions in 13- and 14-year-old girls (Bengtsson et al., 2016).

##### Gender (41 studies, 47 tests of association)

Of 47 tests of association that evaluated gender as a risk factor for compassion, 24 (51%) found that females were more likely to be compassionate than males. One such study reported that female, but not male, physicians demonstrated increases in compassion following empathy training (Riess et al., 2012). Twenty-one tests reported no significant differences in compassion by gender. In the one study that reported greater compassion among males, spouses were asked to rate the level of compassion of their spouse; in this case, wives were more likely to rate their husbands as compassionate than *vice-versa* (McDonald et al., 2018).

##### Race and ethnicity (8 studies, 9 tests of association)

In general, race and ethnicity of the compassion-giver were not associated with compassion (5 of 7 associations, 71.4%). However, two studies reported that persons of color were less likely to be offered compassion than Caucasians (as potential receivers of compassion). The race and ethnicity of the study subjects (the ‘compassion-givers’) did not appear to influence this tendency (Stellar et al., 2012; Hirsh et al., 2019).

##### Religiosity, spirituality, and faith (10 studies, 13 tests of association)

In 10 (76.9%) of 13 tests, religiosity and spirituality, defined differently among the studies, were positively associated with compassion. In a survey of psychiatrists, Rindt-Hoffman et al. found a significant positive relationship between spirituality and compassionate love for a specific close other, but not for strangers or humanity in general (Rindt-Hoffman et al., 2019), while Sprecher and Fehr reported that religiosity and spirituality were associated with compassionate love for others, particularly for strangers and humanity (Sprecher and Fehr, 2005).

##### Socioeconomic status of compassion recipient (4 studies, 4 tests of association)

Of four studies examining the socioeconomic status of the compassion-recipient and whether compassion was offered, one found no association when controlling for perceptions of distress in the recipient (Stellar et al., 2012) and one, in an experimental setting, showed greater compassion for persons with lower income (Delton et al., 2018). However, in healthcare settings, greater physician bias and less compassionate care were reported for patients of lower socioeconomic status (Hirsh et al., 2019), while higher-income patients were more likely to perceive their medical care as compassionate (O’Malley and Forrest, 2002).

##### Country of origin (9 studies, 10 tests of association)

Ten tests examined the relationship between compassion and study participants’ country of origin. Patterns for specific countries were inconsistent and inconclusive. Chang et al. reported empathy being higher in subjects from South America than Turkey, although scores for alleviating suffering were highest in Turkey and lowest in South America (Chang et al., 2021). Gilbert et al. (2017) found that compassion for others was higher in Portugal than in the United States or the United Kingdom. Mikulincer et al. (2005) reported greater compassion among study participants in the United States than in Israel. In a study by Howick et al. (2017), patients rated empathy among clinicians using the CARE measure. Patients in Australia, the United States, and the United Kingdom rated their caregivers as highest in empathy; the lowest scores were reported in Hong Kong. Sinclair et al. (2020) reported greater compassion among Spanish participants than their Canadian counterparts.

##### Other demographic factors

Other demographic factors were examined in smaller numbers of studies or had no strong association with compassion, including marital status, education level, home ownership, household size, political affiliation, employment status, and type of healthcare provider (Table 3).

#### Domain 2—Personal characteristics, disposition, and skills

Several studies found significant associations between compassion and personal characteristics or skills of the compassion-giver (Table 3). Perspective-taking and empathic concern, often considered necessary for compassion, were examined in relatively large numbers of studies (7 and 12, respectively). Ten studies also examined the relationship between compassion and dispositional secure or insecure attachment.

##### Perspective-taking (7 studies, 10 tests of association)

Perspective-taking is the cognitive skill of understanding the situations of others (Davis, 1983). Seven studies examined 10 potential associations between compassion and perspective-taking; perspective-taking was a positive independent risk factor for compassion in six associations and an effect modifier in two. In a survey of 202 young adults in New Mexico, Davis et al. (2019) found that perspective-taking positively predicted empathic concern, which in turn, was associated with self-reported prosocial behaviors; perspective-taking was also associated with previous exposure to major stressful life events. In a survey of 201 patients with metastatic cancer in France, patient assessment of physician perspective-taking was positively associated with compassion (Lelorain et al., 2015). An experimental study of undergraduate students by Lim et al. (2015) found that both perspective-taking and empathic concern led to dispositional compassion, which, in turn, predicted compassionate action when confronted with an unwell and overworked confederate. Vollhardt and Staub (2011), also studying undergraduate students, found that perspective-taking mediated the relationship between compassion-givers’ previous experience of suffering and their prosocial attitudes and helping behavior. Cassidy et al. reported positive associations between perspective-taking and compassion, regardless of the degree of similarity between the compassion-giver and the target (Cassidy et al., 2018).

##### Empathic concern (12 studies, 24 tests of association)

Batson defines empathic concern as an “other-oriented emotion elicited by and congruent with the perceived welfare of a person in need” (Batson, 2017). Of 24 tests of association between empathic concern and compassion, 13 (54%) showed a direct effect on compassion and four more (17%) reported empathic concern as a positive modifier. Boulianne et al. (2018), studying the public response to the massive 2016 wildfire in Fort McMurray, Alberta, Canada, reported that concern and professed care for the victims were associated with higher odds of actually helping them. Lim and Desteno (2016) observed that empathic concern, but not perspective-taking, reliably predicted enhanced dispositional compassion. In the study by Davis and colleagues mentioned above, empathic concern provided the link between previous stressful life events and compassionate prosocial behavior (Davis et al., 2019). Cassidy et al. (2018) also reported positive associations between empathic concern and compassion.

Empathic concern appears to moderate the relationship between compassion and some of its risk factors, including adverse life events (Davis et al., 2019), severity of adversity or perceived suffering (Lim and DeSteno, 2020), and target group membership (Tarrant et al., 2009). In this latter study, Tarrant and colleagues found that empathic concern can override the effect of outgroup membership of the compassion target, which is typically associated with decreased compassion. In contrast, Zoghbi-Manrique-de-Lara and Viera-Armas (2019) reported that “common humanity,” but not empathic concern, mediated the link between ethical organizational leadership and compassionate actions among peers within the organization. Similarly, Cialdini et al. (1997) reported that the association between empathic concern and helping behavior became non-significant when “oneness”—a measure of perceived self-other overlap—was considered.

##### Secure attachment (dispositional; 2 studies, 4 tests of association)

Two studies reported positive associations between compassion and general measures of attachment security. Shiota et al. examined this association in the context of adult romantic relationships (Shiota et al., 2006). Rindt-Hoffman et al. (2019) reported that secure attachment was associated with compassionate love for close others and a specific close other, but not for strangers or humanity in general, suggesting that the effect of attachment may depend on the target of compassion.

##### Attachment avoidance (dispositional; 9 studies, 11 tests of association)

Nine studies measured attachment avoidance using a subscale of the Experience in Close Relationships questionnaire. Of 11 tests of association, 10 (91%) reported significant *negative* associations with compassion. Sabey and Rauer found that among older heterosexual married couples, wives’ attachment avoidance was predictive of less self-reported compassionate love for husbands a year later (Sabey and Rauer, 2018). Consistent negative associations have also been reported in experimental settings (Mikulincer et al., 2005; Cassidy et al., 2018).

##### Attachment anxiety (dispositional; 8 studies, 11 tests of association)

In contrast, only one (9%) of 11 tests showed a negative association between dispositional attachment anxiety and compassion. This was reported by Cassidy et al. (2018) in an experimental setting.

##### Self-compassion (5 studies, 5 tests of association)

Of the five tests that examined the relationship between self-compassion and other-directed compassion, four (80%) found a significant positive association. Bengtsson et al. (2016) highlighted the importance of “the perspective-taking component of self-compassion,” while Henshall et al. correlated self-compassion with both compassion for others and compassion at the organizational level (Henshall et al., 2018). Jazaieri and colleagues found that compassion training strengthened the association between caring for self and caring for others (Jazaieri et al., 2016).

##### Social and emotional intelligence (3 studies, 3 tests of association)

Social and emotional intelligence, a construct related to empathic concern, was significantly associated with compassion in all three studies in which it was examined. In a quasi-randomized controlled trial of training to cultivate emotional skills, Paakannen et al. reported a significant association between emotional skills and compassion; the positive effect of training on compassion was mediated by improved emotional skills (Paakkanen et al., 2021). Prabha and Mittal, reporting on a survey of 200 adults in Jaipur, India, found that social intelligence was positively correlated with both altruism and compassion, and negatively correlated with aggression (Prabha and Mittal, 2019). A survey of adults in Canada and Spain strongly linked trait emotional intelligence and emotionality to compassion (Sinclair et al., 2020).

##### Prosocial attitudes and values (3 studies, 3 tests of association)

Three studies that examined positive attitudes towards compassion (Kirby et al., 2021), egalitarian values (Owuamalam and Matos, 2019), or self-transcendent values (McDonald et al., 2018) found positive associations with compassion. As defined by McDonald et al. (2018), self-transcendent values are closely related to eudaimonia (happiness arising from fulfilling one’s virtuous potential) and well-being.

##### Well-being/eudaimonia (2 studies, 4 tests of association)

Both studies that examined well-being or eudaimonia reported positive associations with compassion. Using moment-to-moment experience sampling methods, Runyan et al. (2019) found a strong association between eudaimonia and compassion. Eudaimonia was more closely associated with compassion than with empathy. Further, among subjects reporting lower eudaimonia—but not those with higher eudaimonia—as measured by experience sampling, feeling overwhelmed predicted lower moment-to-moment compassion (Runyan et al., 2019). Gilbert et al. (2017), surveying university students in the United Kingdom, Portugal, and the United States, found a weak but significant correlation between well-being and compassion for others.

##### Depressive symptoms (4 studies, 5 tests of association)

Five tests evaluated the association between compassion and depressive symptoms in the compassion-giver, with mixed results. Three studies found no significant correlations between compassion and depression or anxiety (Moore et al., 2015; Gilbert et al., 2017; Lopez et al., 2018). In a survey of more than 1,000 adults ages 55–99 years, neither past or current depression nor anxiety were significantly associated with self-reported compassion for others (Moore et al., 2015). In contrast, using data from the Young Finns Study—a multi-decade longitudinal population-based study of six birth cohorts ranging from 3 to 18 years old at the time of enrollment—Hintsanen and colleagues reported a strong negative correlation between depressive symptoms and self-reported compassion for others; depressive symptoms also attenuated the association between having received parental emotional warmth as a child and self-reported compassion for others in adults (Hintsanen et al., 2019).

##### Physical health (4 studies, 5 tests of association)

Mixed results were observed concerning physical health. Using the Young Finns longitudinal cohort study, Saarinen et al. (2020) found that frequent somatic complaints predicted a slower trajectory of increasing compassion later in adulthood. In a prospective study of older married heterosexual couples, poorer health of the husband predicted increased compassionate love from the wife some 17 months later (Sabey and Rauer, 2018). In a survey of mostly African-American women who were receiving healthcare, 56% who described their health as excellent ranked their physician as compassionate, compared to 39% who described their health as poor to fair (O’Malley and Forrest, 2002). Lopez et al. found no association between the presence of physical disease and self-reported compassion (Lopez et al., 2018).

##### Self-efficacy (4 studies, 6 tests of association)

Two (33%) of six tests of association between self-efficacy and compassion found a positive result. A study by Lim and DeSteno (2020) reported that beliefs about one’s ability to help predicted felt compassion. Other studies found the role of self-efficacy in prompting compassionate action to be affected by the number of “victims” and previous history of adversity (Cameron and Payne, 2011; Lim and DeSteno, 2020).

##### Personality traits (7 studies, 28 tests of association)

Relatively few studies examined prosocial personality traits such as openness, humility, and emotionality, but all of these traits were significantly and positively associated with compassion (Shiota et al., 2006; Krause and Hayward, 2015; Choudhary and Madnawat, 2017a; Krause et al., 2018; Singh et al., 2018). The personality trait of neuroticism was not associated with compassion, while conscientiousness and agreeableness were both associated with compassion in 40% of tests (Shiota et al., 2006; Choudhary and Madnawat, 2017a; Sinclair et al., 2020). Agreeableness was associated with compassion in Canadians, but not Spaniards (Sinclair et al., 2020).

##### Social power of compassion-giver (1 study, 2 tests of association)

Social power—the influence a person exerts over other people as a result of social status or position—was inversely associated with compassion for others in a study by van Kleef and colleagues (van Kleef et al., 2008). Further, individuals of lower social power, but not their higher-power peers, showed a commensurate increase in compassion as severity of suffering and victim distress increased.

##### Burnout (2 studies, 2 tests of association)

A survey of physicians and nurses by Lown and colleagues reported negative correlations between their scores on the Schwartz Center Compassionate Care Scale and how frequently they indicated that burnout inhibited their ability to provide compassionate care (Lown et al., 2019). In contrast, among survey participants recruited through Amazon Mechanical Turk (MTurk), a crowdsourcing marketplace, burnout did not predict scores on measures of compassion or empathy (Kirby et al., 2021). The two studies used different scales to measure burnout, making it difficult to directly compare.

##### Mind wandering to negative (1 study, 1 test of association)

Using experience sampling methods, Jazaieri and colleagues demonstrated that caring behavior was less likely when study participants’ minds wandered to negative or neutral topics (Jazaieri et al., 2016).

##### Other risk factors

Relatively few studies examined other characteristics including resilience, mindfulness, a sense of common humanity, environmental compassion, passionate love, fears of compassion, positive affect, commitment to a career course in university students, studying the sciences, and valuing being well-off financially. Significant positive associations were observed with compassion for some of these characteristics (Table 3).

#### Domain 3—Personal history and experience of the compassion-giver

##### Exposure to stressful life events (9 studies, 13 tests of association)

Nine of the 13 associations that examined the role of previous adversity or stressful life events found a positive relationship with compassion (Vollhardt and Staub, 2011; Moore et al., 2015; Lim and DeSteno, 2016, 2020; Davis et al., 2019). Lim and Desteno reported that compassion was positively associated with severity of past adversity, a relationship that was mediated through increased empathy (Lim and DeSteno, 2016). Vollhardt and Staub found that previous experience of traumatic life events, such as natural disasters or interpersonal and group-based harm, was associated with a significantly greater likelihood of exhibiting prosocial attitudes and helping behaviors for social outgroups experiencing similar adversity (Vollhardt and Staub, 2011).

In a second series of studies, Lim and DeSteno explored the role of previous adversity in moderating the effect of the number of victims on compassionate response. Among persons who had experienced little adversity, compassion tended to decrease with the number of victims, an effect known as the identifiable victim effect (Lim and DeSteno, 2020). In contrast, among those who had experienced previous adversity, compassion increased with the number of victims. However, persons who had experienced previous adversity also expressed greater compassion for single victims than did their low-adversity counterparts. The authors attributed this effect to a greater sense of efficacy (i.e., their perceived ability to alleviate suffering, both for single-victim and group-victim scenarios) in persons who had survived adversity (Lim and DeSteno, 2016).

##### Compassion, empathy, or mindfulness training (14 studies, 15 tests of association)

Twelve (80.0%) of 15 tests that examined the effects compassion, empathy, or mindfulness training showed a significant and positive association with measures of compassion (Table 4). All of them treated compassion as a trait or a skill of individual people. Of the 14 studies, 10 (71.4%) were RCTs (Weibel, 2007; Riess et al., 2012; Jazaieri et al., 2013; Lim et al., 2015; Brito-Pons et al., 2018; Gonzalez-Hernandez et al., 2018; Bas-Sarmiento et al., 2019; Hirsh et al., 2019; Paakkanen et al., 2021); three (21.4%) were longitudinal studies with pre- and post-intervention measures (Jazaieri et al., 2016; Dawson et al., 2021; Vuorinen et al., 2021); and one was cross-sectional in design (Callister and Plante, 2017). Of the 10 RCTs, three tested Compassion Cultivation Training (CCT©; Jazaieri et al., 2013; Brito-Pons et al., 2018); one tested Cognitively Based Compassion Training (CBCT®; Gonzalez-Hernandez et al., 2018); two tested empathy training (Riess et al., 2012; Bas-Sarmiento et al., 2019); and the four remaining tested mindfulness, perspective-taking, emotional skills, or lovingkindness interventions (Weibel, 2007; Lim et al., 2015; Hirsh et al., 2019; Paakkanen et al., 2021). Interventions tested in the three non-randomized studies using a pre-/post-test design included CCT©, Schwartz Rounds, and an intervention focused on compassion and “character strengths” for teachers (Jazaieri et al., 2016; Dawson et al., 2021; Vuorinen et al., 2021). One cross-sectional study tested the association between self-reported compassion in university students and previous participation in a workshop to raise cultural and racial awareness (Callister and Plante, 2017).

**Table 4:** Characteristics of studies that assessed a training intervention to promote compassion.

Type	Author and Year	What tested	Population	Perspective	Results
RCT	Bas-Sarmiento et al. (2019)	Empathy intervention vs. waitlist control	Nursing students, Spain	2^nd^, 3^rd^	Higher post-test scores on compassion with empathy intervention
	Brito-Pons et al. (2018)	CCT© vs. waitlist control	Adults, Chile	1^st^	Improved compassion skills with CCT©
	Brito-Pons et al. (2018)	CCT© vs. MBSR		Adults, Chile	1^st^	Greater compassion with CCT©
	Gonzalez-Hernandez et al. (2018)	CBCT® vs. usual treatment	Breast cancer survivors, Spain	1^st^	Greater total compassion score with CBCT®
	Hirsh et al. (2019)	Virtual perspective-taking intervention vs. control	Resident physicians, United States	1^st^; video simulation	Lower odds of bias (assessed by simulation); increased compassion (self-report)
	Jazaieri et al. (2013)	CCT© vs. waitlist control	Adults, United States	1^st^	Greater compassion in all domains with CCT©
	Lim et al. (2015)	Mobile app and mindfulness training vs. cognitive training	University students, United States	3^rd^	Mindfulness group more likely to give up seat to person who needed it
	Paakkanen et al. (2021)	Emotional skills cultivation training vs. no-intervention control	Workplace managers and employees, Finland	1^st^, 2^nd^	Improved emotional skills, compassion
	Riess et al. (2012)	Empathy training modules vs. standard post-graduate education	Resident physicians, United States	2^nd^	Patients rated intervention group higher on CARE measure
	Weibel (2007)	Lovingkindness vs. no-intervention control	College students, United States	1^st^	Greater increase in compassionate love, but not at 2-month follow-up
Pre-Post test	Dawson et al. (2021)	Schwartz Rounds	Healthcare workers, U.K.	1^st^	No significant effect on compassion score
	Jazaieri et al. (2016)	CCT©	Adults, United States	1^st^ (experience sampling)	No significantly improved self-reported caring behaviors
	Vuorinen et al. (2021)	Character strength training	Early childhood development teachers, Finland	1^st^	Improved “sense of compassion” and other measures
Cross- section	Callister and Plante (2017)	Reported attendance at a racial- or cultural-awareness workshop	University students, United States	1^st^	Higher self-reported Santa Clara Brief Compassion score on survey

CBCT, cognitively-based compassion training. CCT, compassion cultivation training. MBSR, mindfulness-based stress reduction. RCT, randomized clinical trial.

Nine of the 14 studies assessed compassion solely from the first-person perspective of the compassion-giver, using self-report measures (Weibel, 2007; Jazaieri et al., 2013, 2016; Callister and Plante, 2017; Brito-Pons et al., 2018; Gonzalez-Hernandez et al., 2018; Dawson et al., 2021; Vuorinen et al., 2021). Five of the RCTs included assessments from other perspectives (Riess et al., 2012; Lim et al., 2015; Bas-Sarmiento et al., 2019; Hirsh et al., 2019; Paakkanen et al., 2021). Hirsh et al. (2019), in an RCT of a perspective-taking intervention, assessed the effect of training on bias among resident physicians using patient simulation videos (third-person). Riess et al. (2012) used the CARE scale for patients to assess compassion in physicians who had been randomized to receive empathy training modules or other post-graduate training (second-person). Bas-Sarmiento and colleagues (2019) evaluated the effects of an empathy training intervention in nursing students by observing their interactions with actors posing as patients (third-person) and by having those actors rate the interactions using the CARE scale (second-person). Lim and colleagues tested app-based mindfulness training using a third-person behavioral measure (Lim et al., 2015). While subjects waited in an area outside the experimental laboratory, a confederate entered using crutches, wearing a walking boot, and obviously in discomfort. A compassionate response was defined as the subject standing and offering his or her seat to the confederate. Finally, in the workplace setting, Paakanen et al. evaluated the impact of training organizational managers to cultivate emotional skills, based on employees’ assessments of compassion in their managers (second-person; Paakkanen et al., 2021).

Two studies, both pre-/post-test in design, did not show a significant positive association between empathy or mindfulness training and compassion (Table 4). Dawson et al. found no significant increase in self-reported compassion among UK healthcare workers who regularly attended Schwartz Rounds over an eight-month period (Dawson et al., 2021). Jazaieri et al., using experience sampling, found a positive, but non-significant trend in the proportion of times persons receiving CCT© reported caring behaviors (Jazaieri et al., 2016). Finally, Weibel showed a significant difference in self-reported compassionate love between intervention and control groups immediately following four weekly 90-min sessions of loving-kindness meditation training, but this difference attenuated and was non-significant at the two-month follow-up assessment (Weibel, 2007).

In addition to improved compassion, many of the studies on training also reported improvements in empathy, well-being, relational skills, and other desirable outcomes.

##### Parental warmth and acceptance (1 study, 2 tests of association)

Few studies in our sample explored the importance of secure attachment during childhood in relation to one’s compassion later in life. One study, by Hintsanen et al., found that parental warmth in childhood was positively associated with compassion in adulthood (Hintsanen et al., 2019).

##### Other historical factors

Other potential historical or experiential risk factors for compassion included childcare environment, participating in a cultural immersion trip during college, and length of time practicing medicine. These factors were examined in only a few studies (Table 3).

#### Domain 4—Habitual behaviors of the compassion-giver

##### Church attendance (5 studies, 7 tests of association)

Of seven tests of association between church attendance and compassion, four were significantly and positively associated (Sprecher and Fehr, 2005). However, Krause and Hayward (2015) reported that religious commitment, but not church attendance, was associated with compassion.

##### Other behavioral factors

Relatively few studies assessed other behavioral traits or habits of the compassion-giver. In the wake of the Fort McMurray wildfire in Alberta, Canada, Boulianne and colleagues found that those who used social media were significantly more likely to know someone who was affected, and those who followed the wildfire on social media were nearly twice as likely to help as those who did not follow the fire on social media (Boulianne et al., 2018). Callister and Plante, studying compassion in university students, reported that volunteering and doing community service were highly correlated with self-reported compassion (Callister and Plante, 2017). Lovette-Colyer reported similar findings among students who volunteered for community service, although he found an inverse correlation with compassion for students who were *required* to participate in service learning (Lovette-Colyer, 2013). Both groups of investigators in these latter two studies reported inverse correlations between self-reported compassion for others and partying behavior or participation in college sororities or fraternities (Lovette-Colyer, 2013; Callister and Plante, 2017).

#### Domain 5—Circumstantial or contextual factors related to the compassion encounter

Twenty-three risk factors were examined that relate to the immediate circumstances in which suffering presents the opportunity for compassion. These have been grouped into the following categories: (1) perceptions of suffering and of potential outcomes of compassionate action; (2) relational aspects between the person suffering and the compassion-giver; (3) the inner emotional state of the compassion-giver; (4) time-related considerations; and (5) other risk factors.

##### Perceptions of suffering and outcomes of action

*Severity of suffering (4 studies, 6 tests of association).* Delton and colleagues reported two studies in which “absolute need,” as measured by financial poverty of the victim, was positively associated with compassion (Delton et al., 2018). Cialdini et al. (1997) confirmed this association in an experimental setting and found that in higher-need (i.e., more severe) situations, relational closeness between compassion-giver and the target led to greater empathic concern and willingness to help.

*Chronicity of suffering (1 study, 2 tests of association).* Butts et al., defining chronicity as the “likelihood that the suffering will continue or recur,” found no significant association between chronicity of suffering and helping responses (Butts et al., 2019).

*Number of victims (6 studies, 11 tests of association).* In a meta-analysis of 41 studies, Butts et al. reported that larger victim group size negatively affects both helping intent and helping behavior, a phenomenon known as “numeracy bias” (Lim and DeSteno, 2020) “compassion collapse” (Cameron, 2017), or “identifiable victim effect” (Butts et al., 2019). This effect appears to be influenced by several factors. For example, Lim and DeSteno (2020) reported that persons who had experienced adversity reported significantly *greater* compassion as the number of victims increased, an effect that was modulated by greater self-efficacy in those who had experienced adversity. Cameron and Payne (2011) found that numeracy bias is also influenced by whether the compassion-giver expected to be asked to help; this expectation was not a significant factor for the single-victim condition but it made helping less likely if subjects expected to be asked to help for an eight-victim condition. Finally, Butts et al. (2019) reported that the negative relationship between victim group size and helping intent was stronger when threat severity and certainty of harm were higher.

*Perceived distress in the person suffering (the target of compassion; 2 studies, 3 tests of association).* The compassion-giver’s perception of distress in the person suffering is related to the notion of severity of suffering. Two studies found a positive association between perceived distress and compassion, but in both studies, this effect was attenuated by increased social class or power of the compassion-giver. In an experimental setting, Stellar and colleagues found that subjects of lower social class perceived greater distress in colleagues being subjected to a difficult job interview, which predicted a compassionate response (Stellar et al., 2012). Van Kleef et al. (2008) paired undergraduate students, one of whom would describe an experience that had caused them suffering. Listeners with a higher sense of personal power experienced less distress and less compassion in listening to the accounts of their colleagues than did those with a lower sense of power.

*Perceived positive impact (1 study, 1 test of association).* Butts et al. (2019) found that the compassion-giver’s perceived impact of intervening to reduce suffering—a construct that may be related to self-efficacy—was positively associated with both empathic concern and with helping behavior.

*Perceived personal cost (1 study, 2 tests of association).* In contrast, Owuamalam and Matos (2019) reported that study subjects were more likely to provide assistance when the political cost was low. Their willingness to help when the political cost was high was influenced by the status of the victim; study participants were more likely to assist high-status victims than low-status victims.

*Perceived certainty of harm (1 study, 1 test of association).* Butts et al. (2019) showed that certainty of harm modified the relationship between victim group size and both helping intent and behavior. The negative relationship between victim group size and helping intent was stronger when certainty of harm was higher.

##### Relational factors

*Perceived similarity/in-group (6 studies, 8 tests of association).* All six studies that examined similarity between the compassion-giver and the person suffering observed positive associations with compassion. University students listening to another student describe a distressing experience reported stronger empathy and intention to help if both students belonged to the same university (Tarrant et al., 2009). Valdesolo and DeSteno (2011) showed that experimentally-induced synchronous movement led to perceptions of similarity between pairs of individuals, which were further associated with compassion and altruistic behavior. Vollhardt and Staub found that prosocial attitudes toward tsunami victims were highest among those who had, themselves, suffered from natural disasters (Vollhardt and Staub, 2011). Cialdini et al. (1997) reported that the experience of “oneness” with the target significantly increased both empathic concern and helping.

*Liking/appreciating/valuing the other (2 studies, 2 tests of association).* This construct is closely linked to perceived similarity and relationship closeness. However, in the study by Valdesolo and DeSteno, although synchronous movement increased both the subject’s perceived similarity with and liking for the victim, increased liking was not associated with compassion or helping (Valdesolo and DeSteno, 2011). In an organizational study by Moon et al., employees’ appreciation for their organization’s corporate social responsibility positively influenced their affective commitment to the organization, which, in turn, was associated with expressions of compassion at work (Moon et al., 2014).

*Relationship closeness/psychological proximity (6 studies, 6 tests of association).* Six studies examined the psychological closeness of the compassion-giver and receiver, five finding a positive association with compassion (Cialdini et al., 1997; Mikulincer et al., 2005; Boulianne et al., 2018), and the other finding no significant correlation (Cameron and Payne, 2011).

*Expectation to donate (1 study, 2 tests of association).* Under experimental conditions, Cameron and Payne found that participants’ expectation that they would be asked to provide help to either single or multiple victims favored compassion toward a single victim. By removing this expectation, compassion was significantly more likely to be expressed for multiple victims (Cameron and Payne, 2011).

*High social status of the victim (2 studies, 2 tests of association).* Stellar et al. (2012) found no relationship between social class of an experimental subject undergoing a stressful interview and compassion reported by their peer study partner. In contrast, Owuamalam and Matos (2019) found that when the political cost of compassion was low, egalitarians displayed greater compassion towards higher-status victims and anti-egalitarians had similar levels of compassion for both high- and low-status victims. These findings suggest that when the cost of compassion is perceived to be low, egalitarians can favor the privileged and anti-egalitarians can act equitably.

*Perceived worthiness (1 study, 1 test of association).* Owuamalam and Matos also found that the worthiness that anti-egalitarians assigned to high-status individuals explained their tendency to preferentially offer them help (Owuamalam and Matos, 2019). However, this was influenced by the perceived political cost of helping.

*Diffusion of responsibility (1 study, 1 test of association).* Diffusion of responsibility refers to the perception that responsibility for responding to suffering is shared among many individuals or groups. A study by Cameron and Payne reported that diffusion of responsibility did not play an important role in compassionate responses to incidents with multiple victims (Cameron and Payne, 2011).

##### Inner state of the compassion-giver

*Personal or empathic distress (15 studies, 18 tests of association).* In all five studies by Mikulincer et al. (2005), the compassion score among participants was significantly but not strongly associated with their personal distress. Interestingly, personal distress was consistently associated with attachment anxiety, which was not associated with compassion or helping. In contrast, two studies found no association between personal distress and either prosocial attitudes or helping behavior (Vollhardt and Staub, 2011; Kirby et al., 2021). In experimental settings, Cassidy et al. (2018) found no significant association between distress and compassion, while Cialdini et al. (1997) reported that personal distress and sadness attenuated the relationship between empathic concern and helping. Van Kleef and colleagues reported that among compassion-givers with a low sense of social power, personal distress was positively related to compassion, whereas among compassion-givers with high social power, personal distress was negatively related to a compassionate response (van Kleef et al., 2008).

*Secure attachment (situational; 7 studies, 9 tests of association).* Mikulincer et al. (2005) used implicit and explicit priming techniques to experimentally induce or boost a sense of secure attachment. In all five studies, these techniques were shown to foster both compassion and altruistic behavior. Similar results were found by Cassidy et al. (2018).

*Anticipated positive affect (3 studies, 5 tests of association).* Anticipated positive affect reflects anticipated feelings about how the compassion-giver will feel by rendering assistance. Butts et al. (2019) found significant effects of anticipated positive affect on helping behavior and empathic concern. In a study examining a closely related construct of anticipated “egoistic payoff” of helping behavior, Mikulincer et al. reported a positive association between compassion and the anticipation of “empathic joy” (Mikulincer et al., 2005).

*Recalling vulnerability (2 studies, 3 tests of association).* In two experimental studies, Cassidy et al. (2018) randomized subjects to remember either a time someone close to them hurt their feelings (hurt feelings memory), which they hypothesized would provoke attachment anxiety, or a neutral memory. The hurt feelings memory did not have a significant main effect on compassion.

##### Sense of time

*Being rushed or too busy (1 study, 1 test of association).* In a randomized experiment of seminarians at Princeton Theological Seminary, Darley and Batson found that a sense of being rushed strongly predicted they would not stop to offer assistance to a man (a confederate) lying in an alley in distress (Darley and Batson, 1973). Interestingly, having received an assignment to prepare a talk on the Good Samaritan that same day, a classic Christian parable of compassion for a stranger, was not associated with stopping to offer assistance.

*Length of clinical consultation (2 studies, 2 tests of association).* In healthcare settings, longer consultations with patients were associated with higher patient-reported CARE scores (Lelorain et al., 2015; Howick et al., 2017).

##### Other risk factors

*Oxytocin (1 study, 1 test of association).* Palgi and colleagues found that dosing subjects with oxytocin increased compassion when the target of compassion was a woman but not a man, irrespective of the gender of the compassion-giver (Palgi et al., 2015).

*Being at home (1 study, 1 test of association).* Using experience sampling, Runyan and colleagues found greater levels of compassion when the study subjects were at home, as opposed to outside, in class, or at work or school (Runyan et al., 2019).

#### Domain 6—Organizational and structural factors

##### Social and organizational support (3 studies, 3 tests of association)

In a cross-sectional study of university students, Beutel and Marini found that compassion was positively associated with social support, conceptualized as having “someone I can turn to if I need help” or “someone I can talk to, if I need to” (Beutel and Marini, 1995). Lown et al. reported that, among nurses and physicians, compassion-related behaviors were inversely correlated with a lack of perceived organizational support (Lown et al., 2019). In another study by Lown et al., perceptions of organizational support were positively associated with nurses’ assessment of their own compassionate care (Lown et al., 2020).

##### Ethical and compassionate leadership (3 studies, 3 tests of association)

A longitudinal study in the public service workplace by Eldor reported that employees’ perception of having received compassion from supervisors at baseline predicted improved employee engagement, lower burnout, and organizational citizenship behavior during the follow-up assessment, as well as employee service-oriented performance and compassionate behavior toward clients (Eldor, 2018). Other investigators reported a positive association between ethical leadership and peer-focused organizational citizenship behavior, which was mediated through a sense of common humanity (Zoghbi-Manrique-de-Lara and Viera-Armas, 2019). Among a diverse group of businesses in South Korea, perceptions of corporate social responsibility were positively related to compassion at work (Moon et al., 2014).

##### Organizational compassion (3 studies, 3 tests of association)

Henshall et al. (2018) found perceived organizational compassion to be significantly associated with employees’ compassion for others. In the healthcare setting, Lown et al. found positive correlations between nurses’ perceived organizational compassion scores and self-reported scores for their own compassionate caregiving (Lown et al., 2020). Moon and colleagues reported that employee compassion was positively related to the employees’ perception of their organization’s social engagement as being just and compassionate (Moon et al., 2014).

##### Perceived organizational threat (2 studies, 2 tests of association)

Perceived organizational threat—i.e., workplace-related stresses, challenges, and threats—showed a weak negative correlation with employees’ compassion for others in a study by Henshall et al. (2018). This association was no longer significant in a follow-up study when controlling for self-compassion, perceived organizational compassion, and gender.

##### Belonging to a supportive organizational unit (1 study, 1 test of association)

In the healthcare setting, Lown et al. found that having a caring nursing team (distinguished from the organization as a whole) was strongly and positively associated with nurses’ perceptions of organizational compassion and with their self-reported individual compassion scores (Lown et al., 2020).

##### Organizational aspects of healthcare (1 study, 3 tests of association)

In a survey of mostly African-American women, O’Malley and Forrest (2002) found that their perception of compassion in primary care physicians was associated with higher organizational health care accessibility, continuity of care, and coordination of specialty care, but not with geographic or financial accessibility. The authors reported that women who highly rated their doctor’s ability to address their health care needs also rated them as highest in compassion.

## Discussion

Understanding the epidemiology of compassion—how and why it is clustered—could help inform and guide efforts to promote compassion at individual and societal levels. The current review attempts to summarize the quantitative scientific literature on factors associated with compassion.

### Challenges and limitations

Several challenges were encountered. First, the scientific literature on compassion is scattered across many disciplines, each with its own methods and conventions. The concepts, definitions, and measures of compassion differ across disciplines and even among investigators within the same discipline (Strauss et al., 2016; Mascaro et al., 2020). Relatively few studies evaluate compassion as an outcome using quantitative data. In addition, there is little standardization across studies regarding the concepts and definitions of potential risk factors for compassion, or the statistical methods used to test for association with compassion. Such heterogeneity precluded the possibility of a meta-analysis and made it difficult to summarize measures of effect for specific risk factors.

Second, as Joan Halifax notes, compassion is not a single, easily defined entity, but rather is comprised of non-compassion elements (Halifax, 2012). We were guided by a simplified model of compassion that includes three fundamental elements: cognitive appraisal (awareness of suffering); empathy (emotional resonance with the person suffering); and action (or at least the intent of acting) to alleviate suffering or its causes. Considerable scientific research now exists on attributes or skills that are thought to foster (and, in some cases, be manifestations of) compassion, such as perspective-taking, empathic concern, altruism, and prosociality—each with their own emerging literature of associated correlates and risk factors. We focused our review on a construct of compassion that involves both empathy and intention to act. In doing so, we undoubtedly excluded articles that address less direct (although important) precursors of compassion (e.g., factors that promote perspective-taking or empathy).

Third, the relatively poor quality of the data and the high proportion (39%) of studies that used a cross-sectional design make it difficult to infer causality. Self-report measures—which may or may not relate to actual behavior—were used in 87% of studies. Further, with the exception of experimental studies in psychology laboratories (e.g., Cialdini et al., 1997; Mikulincer et al., 2005; Tarrant et al., 2009; Lim and DeSteno, 2016, 2020; Cassidy et al., 2018), few studies adequately controlled for potential confounders or analyzed data for factors that might modify relationships between reported risk factors and compassion (i.e., effect modifiers). Thus, a more nuanced set of studies is needed that includes adequate analysis of multiple covariates and controls for the influence of known risk factors.

Fourth, the geographic representativeness of the studies in this review is limited. More than half of the studies were conducted in North America, and university students comprised the majority of participants. Relatively few studies included subjects from Africa or South America.

### Patterns

With these limitations in mind, several overall patterns emerged. Current quantitative research on compassion overwhelmingly focuses on individual persons and their capacity to give compassion to others (Figure 2). Few studies in our review explored capacities or barriers to *receiving* compassion. Pioneering work by Gilbert and others on “fears of compassion” begins to address these barriers (Gilbert et al., 2011; Asano et al., 2017; Kirby et al., 2019); this research has important implications for human flourishing (Gilbert, 2020). For example, a recent study by Ramalho et al. highlighted the significant role of receiving compassion in improving quality of life among persons with chronic disease (Ramalho et al., 2021). New measures, such as the Compassion Engagement and Action Scale, include sub-scales on receiving compassion (Henje et al., 2020).

Recent work by a growing number of investigators has focused on the role and importance of compassion within organizations (Worline and Dutton, 2017). Most of these studies have been qualitative, rather than quantitative, in nature, and address themes such as compassion fatigue, compassion satisfaction, or burnout. Relatively few focus on other-directed compassion as an outcome. Nonetheless, several articles in this review underscore the importance of organizational culture, leadership, social support, and commitment to ethical principles for nurturing compassion among employees. In healthcare settings, organizational commitment to person-centered care, including coordination, continuity, and accessibility were positively associated with perceptions of compassion among patients (O’Malley and Forrest, 2002).

### Implications for an epidemiology of compassion

Compassion is enacted in particular times and places, by particular people, and is influenced by social, cultural, and organizational norms as well as by the physical environment. To explore the implications of the risk factors identified in this review for an epidemiology of compassion, we consider them in the context of three traditional parameters of descriptive epidemiology: person, time, and place. In this framework, compassion can be considered a characteristic or capacity of an individual person (i.e., a host factor). It can also be affected by time (e.g., with age) or one’s perception of time (e.g., feeling rushed), and it varies by place (i.e., particular physical or social environments; Figure 2). These three parameters overlap and interact.

#### Person (host factors)

As noted, most of the studies that met our inclusion criteria treated compassion as a host factor (i.e., a characteristic of an individual human being that predisposes them to respond to suffering with compassion; Figure 2). These host factors include demographic features; personal characteristics, dispositions, and skills; personal history and experience; and habitual behaviors. With the above limitations of available research in mind, several key signals emerged in the data, some of which point to modifiable risk factors. What follows is a discussion of the implications of these signals, by domain.

##### Domain 1—Demographic factors

*Gender*. The finding that female gender was significantly associated with compassion in the majority of studies that evaluated this variable aligns with the perception that women are more compassionate than men. This finding, which investigators did not explore further, is likely influenced, at least in part, by gendered social norms.

*Age*. Although the relationship between compassion and age was mixed, we observed a general trend among these studies, in which compassion increased with age during mid-adulthood. The meaning and reason for these observations have not been adequately explored.

*Religiosity and spirituality.* Religiosity and spirituality were associated with compassion in 10 (76.9%) of 13 tests. Religious scholar Karen Armstrong describes compassion as a common thread across all major religions and spiritual traditions (Armstrong, 2009). Unfortunately, religion also has the power to divide, and in some cases, justify cruelty and the withholding of compassion for out-groups (e.g., members of minority sects or persons with other religious backgrounds). In an increasingly pluralistic and interconnected world, the role of religion in fostering compassion—particularly for the stranger and the “distant other”—requires greater attention.

##### Domain 2—Personal characteristics, disposition, and skills

Many of the personal characteristics that were most strongly associated with compassion are considered precursors to, or elements of, compassion. Social and emotional intelligence and perspective-taking facilitate the recognition of suffering in others. Empathic concern activates the emotional resonance that prompts the desire to alleviate suffering. The most common approaches to standardized compassion training incorporate elements of perspective-taking, empathic concern, intention, and self-compassion. The positive findings from the studies evaluating such training—including all 10 RCTs—point to the importance of these elements for cultivating compassion. In some models of compassion, intention is considered essential (Worline and Dutton, 2017). Intention is shaped by prosocial attitudes and values, which were associated with compassion in all three studies that examined them (McDonald et al., 2018; Owuamalam and Matos, 2019; Kirby et al., 2021).

*Attachment.* Attachment theory has proven to be a powerful framework for understanding the nexus of safety, caregiving, and compassion (Mikulincer et al., 2001, 2005; Gilbert, 2020). Secure attachment as a trait emerged as a strong and consistent risk factor for compassion in the studies we reviewed. Although established in childhood and modified by life experience, secure attachment has life-long effects, influencing empathic concern in preschool-age children (Murphy and Laible, 2013), the development of moral emotions (Costa Martins et al., 2021), the ability to provide empathic support to peers during the teenage years (Stern and Cassidy, 2018), and the quality of adult relationships (McGinley and Evans, 2020). Research by Mikulincer et al. (2005) and Cassidy et al. (2018) demonstrated how attachment can be primed experimentally by imagining the presence of a secure, nurturing other. This approach warrants further attention for efforts to develop compassion in situations where individuals feel insecure or under threat, but desire to respond with compassion.

The Experience in Close Relationships questionnaire, used in most of the studies that assessed attachment, includes subscales for attachment anxiety and attachment avoidance. Consistent with the broader literature (Mikulincer et al., 2001, 2005), the studies we reviewed reported that attachment avoidance was strongly associated with lower compassion scores, whereas attachment anxiety was associated with self-focused distress, but not with other-oriented compassion. Addressing attachment avoidance is a central component of compassion-focused psychotherapy (Gilbert, 2020).

*Self-compassion*. Self-compassion was associated with other-directed compassion in four of the five studies that examined this relationship. The nature of this relationship is complex and controversial (Strauss et al., 2016). The cross-sectional design of these studies makes it difficult to draw causal inferences.

*Power*. The negative relationship between social power and compassion aligns well with observations in many organizational and political settings, and points to an urgent need to cultivate compassion among leaders and those with influence.

##### Domain 3—Personal history and experience

Two factors related to the history or experience of the compassion-giver emerged as particularly important: compassion training and previous experience of suffering or adversity.

*Training*. Intentional training to improve one’s capacity for compassion was well-represented among the intervention studies that met our criteria for inclusion. It was also the most rigorously evaluated; 10 of 14 such studies were RCTs. Recent advances in neuroscience have documented brain plasticity and the human capacity to change one’s response to suffering (Davidson and McEwen, 2012; Weng et al., 2013). Emerging evidence indicates that different forms of contemplative training have different effects and that practices can be tailored to strengthen specific compassion-related skills (Singer and Engert, 2019). These findings suggest that expansion of opportunities for intentional training will be important for compassion to flourish at the societal level.

*Previous adversity.* Previous experience of suffering was consistently associated with compassion, a finding that supports the theoretical framework known as “altruism born of suffering” (Vollhardt and Staub, 2011). This finding is also consistent with the enactive view of compassion proposed by Halifax, which posits that memory is important for the emergence of compassion (Halifax, 2012). Empathic concern, which can be enhanced through the experience of adversity, may be an important mediator between previous adversity and compassion (Davis et al., 2019).

The relationship between suffering and compassion is paradoxical. As a virtuous response, compassion seeks to alleviate suffering, yet, as these studies show, the experience of suffering, itself, can predispose humans to respond compassionately to the suffering of others. The experience of suffering can also lead to its perpetuation (Basto-Pereira et al., 2022). Understanding how and under what conditions suffering leads to post-traumatic growth and meaning-making that foster compassion for others is an important area for further work.

#### Time

Relatively few studies addressed the epidemiologic dimension of time. Two longitudinal studies suggested that compassion increases from young-adulthood into middle-age (Hintsanen et al., 2019; Saarinen et al., 2020), but two others reported decreases in compassion within a two-year period among older married couples (Sabey and Rauer, 2018) and adolescents (Bengtsson et al., 2016). These decreases were attributed not to time itself, but to other factors, i.e., attachment avoidance and negative self-perception, respectively.

Using experience sampling methods, investigators have begun to explore moment-to-moment variability in compassion as an ephemeral state, rather than a relatively stable trait (Jazaieri et al., 2016; Runyan et al., 2019). Additional work is needed to understand the patterns, causes, and consequences of these fluctuations.

The *perception* of time seems to strongly influence whether one responds to suffering with compassionate action; feeling rushed or “time-compressed” is associated with decreased likelihood of helping behavior (Darley and Batson, 1973). Lack of time is consistently cited by healthcare providers and global health professionals as a major barrier to compassionate care and compassionate leadership, respectively (Babaei and Taleghani, 2019; Harrel et al., 2021). Patients’ perception of their healthcare providers’ compassion is associated with the length of clinical consultation (O’Malley and Forrest, 2002). An encouraging study by Fogarty et al. suggests that compassion can be communicated in healthcare settings even when time is severely constrained (Fogarty et al., 1999).

#### Place (physical and social environment)

Available data also suggest that compassion is influenced by physical, social, and organizational environments. All 10 tests for association that examined the relationship between compassion and country of residence—a crude spatial indicator—found national differences, although the direction of these differences was inconsistent with respect to specific countries.

As with the dimension of time, experience sampling methods reveal intriguing differences in the moment-to-moment experience of compassion associated with specific places. For example, Runyan et al. (2019) found greater levels of compassion when study subjects were at home, as opposed to outside, in class, or at work or school. One might speculate that the spaces in which one feels more secure, safe, and supported are more conducive to compassion.

Compassion in humans evolved among small groups in specific places. The role of place and geographic proximity in nurturing compassion has changed radically with rapid advances in communications technology and global travel. Extending compassion to the abstract population level, as is required in the field of global health, for example, requires new ways of imagining ourselves in relation to distant others who may be suffering.

Several of the organizational studies in this review highlight the importance of social norms and organizational culture in creating the conditions in which compassion can emerge. These studies underscore the importance of local, socially-relevant environmental factors in nurturing or inhibiting compassion and point to the potential of further research using the tools and methods of environmental epidemiology.

#### Other considerations

##### Moment of the compassion encounter

Risk factors related to person, time, and place all appear to influence the moment in which suffering is apprehended and compassion emerges. Some of these risk factors are related to dispositional host factors, such as capacity for perspective-taking, social and emotional intelligence, and empathic concern. Others are related to the particularities of the suffering itself, such as its severity and the number of victims. Additional risk factors are rooted in the relationship between the person in the position of offering compassion and the person suffering, such as perceived in-group similarity and psychological proximity. In addition, factors related to the inner emotional state of the compassion-giver—such as emotional distress, a sense of secure attachment, and feeling rushed—play important roles in determining the probability of a compassionate response. Several studies illustrated the interconnected and interdependent nature of these and other factors at the moment of encounter.

Investigators have explored the underlying dynamics at the moment of encounter through different lenses. Loewenstein and Small focused on the interaction between sympathy, “which is caring but immature and irrational” and deliberation, “which is rational but uncaring” (Loewenstein and Small, 2007), while Poulin (2017) has explored the importance of intention and self-related goals in moving from deliberation to compassionate action. The appraisal model of compassion proposed by Goetz and colleagues illuminates both conscious and subconscious factors that determine whether witnessing negative outcomes leads to compassion (Goetz et al., 2010). Further research is needed to understand the degree to which factors associated with the withholding of compassion at the moment of encounter can be overcome.

##### Compassion as a transmissible agent

In addition to considering the dimensions of time, person, and place, infectious disease epidemiology focuses on transmission dynamics of the infectious agent. None of the studies overtly approached compassion as a transmissible agent, applying the tools and approaches of infectious disease epidemiology. However, studies of organizational compassion provide clues as to the potential of this approach. For example, in a longitudinal study in Israel, Eldor examined “public service sector employees who receive compassionate feelings such as affection, generosity, caring, and tenderness from their supervisors” (Eldor, 2018). Receiving compassion from supervisors at the beginning of the study significantly increased subsequent employee compassion for others, as measured by organizational citizenship behavior and employees’ compassionate behavior toward clients (as assessed by clients). Similarly, it is possible to view secure attachment in adulthood having been “transmitted” by parents during early childhood. Kirby et al. have explored how compassion “flows,” underscoring the positive role of attachment as well as factors that inhibit this flow, such as fears of compassion (Kirby et al., 2019). The maturation of thought and scholarship on fears of compassion provides a foundation for understanding factors that promote and inhibit transmission of compassion (Gilbert, 2020).

### Implications for research

Despite several limitations, the current examination of existing knowledge and knowledge gaps can help inform a research agenda to better understand the epidemiology of compassion. The diverse risk factors identified in this review point to the complexity with which “non-compassion elements” come together to allow compassion to emerge. The causal pathways leading from suffering to a compassionate response appear to be non-linear and complex. Further, many factors (acting as effect modifiers) appear to be permissive of—or essential for—the arising of compassion in certain settings or in certain populations, but not others.

It is therefore not surprising that some, but not all, studies of a particular risk factor (e.g., gender) showed significant associations with compassion. It is not clear whether such discrepancies are related to differences in study design, definitions, or methods, or rather to variation in the patterns of interplay among “non-compassion elements” in specific contexts. As much as possible, future research on compassion should take into account the contextual factors and the various ways in which “non-compassion elements,” such as perspective-taking, awareness, empathic concern, and memory, are active in particular settings. In addition, the role of risk factors identified in this review (whether as primary causes, confounders, or effect modifiers) should be considered in future epidemiologic studies of compassion.

Regardless of the inherent complexity of compassion, RCTs of various versions of compassion training demonstrate that, if committed and interested, individuals can improve their capacity for compassion. Training works, although it is clear that different types of training can produce different outcomes (Singer and Engert, 2019). In addition to programs currently offered to adults, such as CBCT® and CCT©, the principles that underlie these programs—strengthening perspective-taking, encouraging empathy, fostering self-compassion—are increasingly being incorporated into early childhood education as well as primary and secondary schools (Roeser and Pinela, 2014; Jones et al., 2021; SEE Learning, 2022). Additional research is needed to determine the most effective and consequential interventions across the lifespan and in different settings and to better understand the relational factors that contribute to successful training (Condon and Makransky, 2020).

Our review highlights the fact that compassion researchers have regarded the giving of compassion primarily as an individual predisposition, host factor, trait, or skill. Much less is known about factors associated with the capacity to *receive* compassion. Future research should more fully address not only the giving and receiving of compassion, but its experience or phenomenology, which at its deepest level extends beyond the duality of giving or receiving. Recent work by Sinclair et al. (2016) reveals the richly nuanced experience of compassion among palliative care patients. Patients experienced compassion if they perceived virtues such as love, genuineness, honesty, and kindness in the healthcare provider; if the provider created a relational space of engaged caregiving and sought to understand the patient and their needs; and if the provider attended to multiple patient needs—physical, spiritual, emotional, and family-related—both to alleviate the patient’s suffering and promote their well-being (Sinclair et al., 2016). It is at this level of human connection that compassion fosters human flourishing (Larkin, 2016). Newly-developed experience sampling methods and the tools of social neuroscience could provide crucial insights into the momentary experience of compassion and the most important factors and pathways that contribute to it.

Certain signals arising from the data warrant particular attention in further research. Among these are the role of previous adversity in predisposing one to compassion; the transmission and sustenance of a “compassion climate” within organizations (Nolan et al., 2022); and the attenuation of empathy and compassion with social power. Further, more research is needed to clarify the relationship between compassion and burnout, depression, and anxiety, currently represented by few studies and with mixed results. This is especially important for the development of desperately-needed compassion interventions for the public health and healthcare workforce. To this end, research to elucidate compassion dynamics within organizations and systems is also critical.

Additional research is needed on collective compassion and on organizations as the holders and transmitters of compassion. It appears from current research that ethical and compassionate leadership, organizational values, responsible social engagement, and prosocial operating norms have the potential to increase expressions of compassion among employees, both within and beyond the workplace. Understanding the mechanisms involved is important for the scaling-up of compassion from the individual to the collective level. Although RCTs clearly demonstrate the effectiveness of training for individuals who desire to become more compassionate, little is known about how to motivate individuals who have not self-selected to cultivate their own compassion. Further, the long-term effectiveness of compassion training is not well-understood. Organization-level research, particularly within healthcare settings, could help address these gaps.

Descriptive epidemiology typically characterizes phenomena by person, time, and place. Preliminary evidence—as well as human experience—suggests that compassion is clustered with respect to all three of these parameters. Advancing our scientific understanding of compassion will require more extensive discussion and deliberation to address the heterogeneity of methods, measures, and assumptions currently used by compassion researchers and to develop more standardized approaches. Additional reflection is warranted on potential contributions from various methodological and analytic approaches. Epidemiologic approaches that appear most promising, based on our review, include those commonly used for infectious disease (to understand how compassion is transmitted); chronic disease (which deals with multiple risk factors in complex interactions); mental health (which addresses inner states as well as outer manifestations); and environmental health (which examines the confluence of factors in a particular setting). Application of these epidemiologic methods should be informed by insights from other scientific disciplines engaged in the study of compassion, as well as by in-depth dialogue with spiritual and religious traditions.

## Author contributions

DA, HB, AR, and AG contributed to conception and design of the study. HB, AR, SA, EH, and SL performed the literature search and initial entry and analysis of the data. AR, HB, and DA organized and managed the database. AR and DA cross-checked the final database to ensure quality control. DA wrote the first draft of the manuscript. All authors contributed to the article and approved the submitted version.

## Funding

This work was financially supported by the Templeton World Charity Foundation (Donation TWCF0535).

## Conflict of interest

The authors declare that the research was conducted in the absence of any commercial or financial relationships that could be construed as a potential conflict of interest.

## Publisher’s note

All claims expressed in this article are solely those of the authors and do not necessarily represent those of their affiliated organizations, or those of the publisher, the editors and the reviewers. Any product that may be evaluated in this article, or claim that may be made by its manufacturer, is not guaranteed or endorsed by the publisher.
